# Trends in Neonatal Ophthalmic Screening Methods

**DOI:** 10.3390/diagnostics12051251

**Published:** 2022-05-18

**Authors:** Martin Hložánek, Zbyněk Straňák, Zuzana Terešková, Jan Mareš, Inka Krejčířová, Marie Česká Burdová

**Affiliations:** 1 Department of Ophthalmology for Children and Adults, Second Faculty of Medicine, Charles University and Motol University Hospital, V Úvalu 84, 15006 Prague, Czech Republic; zbynek.stranak@fnkv.cz (Z.S.); zuzana.tereskova@fnmotol.cz (Z.T.); jan.mares@fnmotol.cz (J.M.); marie.ceska-burdova@fnmotol.cz (M.Č.B.); 2 Department of Ophthalmology, Third Faculty of Medicine, Charles University and University Hospital Královské Vinohrady, Šrobárova 50, 10034 Prague, Czech Republic; 3 Department of Pediatric Ophthalmology, Faculty of Medicine, Masaryk University and University Hospital, Černopolní 9, 62500 Brno, Czech Republic; krejcirova.inka@fnbrno.cz

**Keywords:** neonatal ophthalmic screening, artificial intelligence, wide-field digital imaging system

## Abstract

Neonatal ophthalmic screening should lead to early diagnosis of ocular abnormalities to reduce long-term visual impairment in selected diseases. If a treatable pathology is diagnosed within a few days after the birth, adequate therapy may be indicated to facilitate the best possible conditions for further development of visual functions. Traditional neonatal ophthalmic screening uses the red reflex test (RRT). It tests the transmittance of the light through optical media towards the retina and the general disposition of the central part of the retina. However, RRT has weaknesses, especially in posterior segment affections. Wide-field digital imaging techniques have shown promising results in detecting anterior and posterior segment pathologies. Particular attention should be paid to telemedicine and artificial intelligence. These methods can improve the specificity and sensitivity of neonatal eye screening. Both are already highly advanced in diagnosing and monitoring of retinopathy of prematurity.

## 1. Introduction

Neonatal eye screening, in general, shall identify the maximum of congenital ocular abnormalities early after the birth and, thus, contribute to the reduction of long-term visual impairment in selected diseases.

The main reason for early detection of congenital ocular pathologies is the development of visual functions begins rapidly from the sixth postnatal week. If a treatable pathology is diagnosed within a few days after the birth, adequate therapy can be indicated to facilitate the best possible conditions for further development of visual functions. It is particularly valid for diseases leading to significant reduction of the light transmittance towards the retina (e.g., congenital cataract, corneal opacity, vitreous hemorrhage).

Other disorders may be part of complex syndromes or systemic diseases and early identification can help with the final diagnosis (e.g., corneal or lenticular changes, retinal pigmentation).

Some disorders only require observation and are often self-limiting (e.g., small retinal or vitreous hemorrhages).

## 2. Full-Term Healthy Newborns

Primary care providers usually provide traditional neonatal eye screening using the red reflex test (RRT). This test has many advantages, as it is cheap, fast, and has a short learning curve. It tests the transmittance of the light through the optical media (cornea, lens, vitreous) towards the retina and the overall condition of the central part of the retina (its ability to reflect light). The RRT is performed in a darkened room using a direct ophthalmoscope. Its light focuses on the infant’s pupil from approximately 30–45 cm. The test is negative if the appearance is symmetric in both eyes and the reflex is round and bright. Any asymmetry, lack of a red reflex or white or dark defects means positivity of the test, and the infant must be referred to the ophthalmologist for further examination [1,2].

Selected diseases detectable by a positive RRT include corneal opacities, congenital cataract, uveitis, vitreous and retinal hemorrhages, persistent fetal vasculature, and familial exudative vitreoretinopathy, retinoblastoma, hamartomas, Coats’ disease, congenital glaucoma, chorioretinal and optic nerve coloboma. Although the timing of detection of some pathologies would not change the visual outcome, some of them are sight- or even life-threatening (retinoblastoma).

### 2.1. Selected Ocular Congenital Diseases

In this section, we shortly discuss most common diseases that can be detected with the neonatal ophthalmic screening and their treatment is time-dependent.

**Figure 1:**
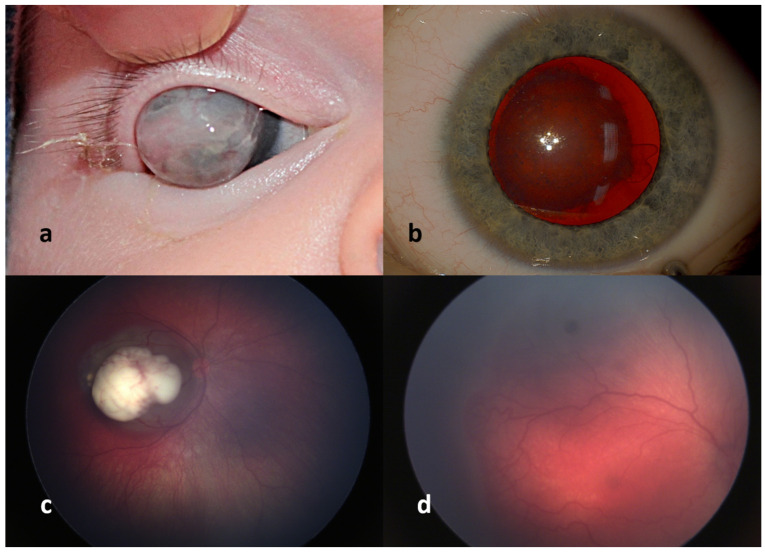
Examples of ocular diseases in infants: corneal dysgenesis (**a**), congenital cataract (**b**), early diagnosed Group B intraocular retinoblastoma (**c**), retinopathy of prematurity stage 2 in zone I and posterior zone II with plus disease (**d**).

Congenital cataract, when optically significant, reduces the amount of light stimulating the retina and leads to profound deprivation amblyopia (Figure 1). This condition requires early surgical removal to allow maximum possible development of visual functions. Correction of aphakia, usually with contact lenses, is necessary after the surgery.

Corneal opacity usually forms part of a complex developmental disorder of the whole anterior part of the eye (dysgenesis of the anterior segment) (Figure 1). The risk of deprivation amblyopia is similar to congenital cataract. However, surgical treatment is much more challenging, as the success rate of corneal transplantation in infants is considerably lower.

Uveitis is the term given to a wide range of intraocular inflammatory diseases. It can involve anterior segment, posterior segment or both (panuveitis). Regardless of its etiology, in case of activity it requires immediate treatment, either causal (infections, for example toxoplasmosis) or symptomatic inhibition of inflammatory activity (both infectious and non-infectious etiology). In case of delay of the treatment, the inflammation may cause irreversible changes like cataract, glaucoma, corneal decompensation, synechiae, traction retinal detachment, macular edema, retinal scars etc.

Retinoblastoma is the most common intraocular malignancy in children. The typical age of diagnosis is 1–2 years of life. Congenital occurrence of this tumor is rare but possible. It can be detected with RRT just in case of central localization (Figure 1). In case of involvement of peripheral retina, only complete retina examination or WFDI is able to detect small tumor. It remains unclear how often would it be diagnosed if every newborn’s complete retina were examined. Some studies report frequency between 0.01% to 0.14% [1].

Congenital glaucoma usually presents with cloudy cornea, photosensitivity, tearing, and rapid and irreversible eyeball growth. It is another example of a disease requiring early diagnosis and subsequent surgery to prevent irreversible damage to ocular tissues.

Retinal or vitreous hemorrhages are quite common finding in newborns and spontaneous resorption is usual. However, if the hemorrhage shades the central part of retina for longer time (weeks), it should be surgically removed due to the risk of deprivation amblyopia.

### 2.2. New Trends in Neonatal Ophthalmic Screening

Despite the undoubted improvement in the early detection of major ocular abnormities by RRT, it is still rather limited in terms of its sensitivity, specificity, and variable quality of screening. Ophthalmologists still encounter a considerable number of patients with congenital diseases that were clearly overlooked in screening tests. RRT is particularly weak in posterior segment (retinal) affections, as it tests reflectivity of a very small retinal area. All peripheral retinal lesions remain, therefore, unrecognized. On the other hand, various anatomical, racial, technical, and other conditions make RRT difficult for non-ophthalmologists, and false-positive screening tests are common [2].

New technologies, such as wide-field digital imaging (WFDI) of the retina, telemedicine techniques, and artificial intelligence (AI), may be beneficial for universal neonatal eye screening [1]. Most pilot programmes have performed neonatal screening using a RetCam Wide-Field Digital Imaging System (Clarity Medical Systems, Pleasanton, CA, USA). The Newborn Eye Screening Test (NEST) study demonstrated the ability of the WFDI system to detect posterior segment abnormalities that were otherwise not identified by standard neonatal RRT [3]. Any posterior segment abnormality was detected with WFDI in every 4th screened infant, potentially sight-threatening abnormality was found in every 7th infant in this study. Most common diseases detectable with the neonatal ophthalmic screening and comparison of suitability of RRT and WFDI shows Table 1.

A telemedicine system with efficient fundus imaging performed by technicians and other health care professionals and reliable, centralized image evaluation systems can mitigate the cost and labor concerns around universal neonatal eye screening.

In addition, the application of AI holds great promise. It can theoretically reduce the need for expert opinions and eliminate potential subjective factors in image evaluation. While AI methods for retinopathy of prematurity (ROP) screening and pediatric cataracts are currently being evaluated, the AI accuracy for such applications would be aided by increasing in the number of (physiological) images available for training [1].

On the other hand, we must keep in mind the relative disadvantages of WFDI as a screening tool. Obtaining images requires pharmacological pupil dilation and administration of anesthetic drops before every session. Compared to RRT, this test is undoubtedly more time consuming and burdensome for the patient; the equipment is by far more expensive, relatively cumbersome, and can be handled only by trained personnel.

### 2.3. Additional Possible Screening Enhancements

Some new possible improvements to other diagnostic schemes have recently emerged in terms of new findings in WFDI screening. For example, the use of WFDI has enabled the identification of a relatively high frequency of retinal hemorrhages (RH) after birth (ranged 0.76–39.36% in different studies) [1]. Although the key factors influencing the RH incidence include the delivery mode, examination techniques, and time of examination after birth, the prognostic markers of severe RH are poorly understood. Therefore, some authors have studied possible biomarkers to better understand disease pathogenesis [4]. It is necessary to conduct further studies to determine modern trends in scientific work in this field. Another area where new guidelines for treatment are emerging is macular hemorrhages (occurring in 0.21–6.02% of newborns in different studies). These could induce visual impairment in some cases and were defined as “referable macular hemorrhages” (RMH). There was consensus that WFDI screening for RMH was a clinically meaningful goal of neonatal eye screening; that management should include observation until resolution or intervention when there was insufficient resolution and/or evidence of visual impairment; and that the screening is safe when performed with contact photography using wide-angle contact cameras, pharmacologic dilation, and photographers with demonstrated ability to obtain the protocol photographs [5].

## 3. Preterm Newborns

Preterm infants form a specific group of newborns due to the risk of retinopathy of prematurity (ROP). ROP is a vasoproliferative disease affecting the development of retinal vascularization in preterm or low-birth-weight infants. Despite all the advances that have been made in diagnosis and treatment, ROP remains one of the leading causes of blindness in children [6,7]. Typically, the nasal and temporal retina is completely vascularized by the 36th and 40th week of gestation, respectively. Any damage to the retinal capillaries during vascularization delays the whole process. The pathophysiology is understood to begin with damage to the developing retinal capillaries (phase one, vaso-obliteration). It could occur prior to or during birth, but it is assumed to occur primarily during the days following delivery. Once the developing vessels are damaged, it is believed that the hypoxic retina responds by producing vascular growth factors stimulating neovascularization (phase two, vaso-proliferation). It may result in successful re-vascularization of the retina (regression of the ROP) or progression to neovascular membranes in the vitreous and subsequent scarring and retinal detachment. Vascular endothelial cell growth factor (VEGF) is one of the most significant growth factors involved [8,9,10].

Screening methods play specific role in ROP, as very small and vulnerable infants require repeated screening tests. That is why considerable attention is paid to the choice of the screening method, as well as to its timing. Both should be strong enough to provide correct staging in time (to start eventual treatment) and regardful to tiny infants at the same time. Therefore, WFDI of the retina using AI is highly advanced in diagnosing ROP.

### 3.1. ROP Risk Factors

Most guidelines use a combination of the primary risk factors—birth weight (BW) and gestational age (GA)—to identify infants who should be screened for ROP [11,12]. Another important risk factor is the oxygen therapy. The administration of supplemental oxygen, its concentration, duration, and prolonged mechanical ventilation belong among the most frequently identified risk factors for severe and treatment-requiring ROP. Other risk factors have also been identified, including maternal factors, prenatal and perinatal factors, demographics, medical interventions, comorbidities of prematurity, nutrition, and genetic factors. Although there are contradictory reports, and the risk may vary between different populations, understanding the ROP risk factors is essential for developing predictive models, gaining insights into pathophysiology of retinal vascular diseases and diseases of prematurity, and determining future directions for treatment and research on ROP [12].

### 3.2. ROP Screening

Current recommendations from the American Academy of Pediatrics, American Academy of Ophthalmology, and the American Association for Pediatric Ophthalmology and Strabismus stipulate that all infants with a birth weight of 1500 g or lower or a gestational age of 30 weeks or less and selected infants with a birth weight between 1500 and 2000 g or a gestational age of more than 30 weeks who are predicted to be at risk for ROP (such as infants with hypotension requiring inotropic support, infants subjected to oxygen supplementation for more than a few days, or infants subjected to oxygen treatment without saturation monitoring) should be screened for ROP [13]. The initiation of acute-phase ROP screening should be based on the infant’s postmenstrual age, i.e., the earlier the infant is born, the longer the time to develop severe ROP. The initial ophthalmic examination should be performed by 31 weeks postmenstrual age or four weeks chronologic age, whichever is the later [14].

### 3.3. International Classification of ROP

The International Classification of Retinopathy of Prematurity (ICROP) established a standard ROP classification. It was originally published in 1984, expanded in 1987, and revisited in 2005 [15]. The third international ROP classification (ICROP3) was published in 2021. It reflects innovations in ophthalmic imaging and novel pharmacologic therapy (e.g., anti-VEGF therapy) with unique regression and reactivation features following the treatment compared to ablative therapy [16]. ICROP 3 holds on current definitions such as zone (location of disease), stage (appearance of disease at the avascular-vascular junction), and circumferential extent of disease. Significant updates in ICROP3 include advanced classification metrics (e.g., posterior zone II, notch, sub-categorization of stage 5, and recognition that there is a continuous spectrum of vascular abnormalities from regular to plus disease).

Prior to the era of anti-VEGF agents, ROP classification focused on acute disease, and less attention was paid to regression. The clinical features and time course of regression after ROP treatment with anti-VEGF differ with laser-treated eyes. When describing later stages of ROP, it is recommended to use the following terms: regression (referring to the disease involution and resolution) and reactivation (referring to the recurrence of acute-phase features). Regression may be complete or incomplete, including the persistence of retinal abnormalities. Regression and reactivation should be considered neither reverse nor recurrence of acute ROP [16].

### 3.4. Predictive ROP Models

Predictive models have been developed to identify high-risk infants and to reduce the number of unnecessary screening examinations. In addition to GA and BW, other factors such as weight gain rate have been incorporated into the models [17,18]. Although these algorithms are not integrated into current screening guidelines, they have been proven to correctly predict treatment requiring ROP while reducing the number of screenings. An ideal ROP screening algorithm must achieve near-100% sensitivity so that not a single case of treatment-requiring ROP is missed. While the existing risk models, such as WINROP [17,18], Co-ROP [19], and CHOP [20], approached this ideal during initial testing, their sensitivity has decreased when applied to other populations, and some cases of infants with severe conditions would be missed if widely used. Some models have demonstrated a reduction in screening burden without missing treatment-requiring ROP in some cohorts [12,21].

Almeida et al. reported a statistically significant difference in placental growth factor (PGF) serum levels at birth and at four weeks between matched infants who did and did not suffer from severe ROP [22]. Silverman et al. reported using plane-wave ultrasound (PWUS) to image, measure, and assess the association of blood-flow velocities in the retrobulbar vessels with ROP stages ranging from stage 0 (immature vessels without ROP) to stage 3. They concluded that ROP stage correlated with flow velocities and proposed blood flow measurement by PWUS as a quantitative, clinically relevant, and easily tolerated means of detecting and assessing the ROP risk in preterm neonates [23]. Also, there are tendencies to identify a possible genetic background of predisposition to ROP [24].

### 3.5. Imaging Systems

#### 3.5.1. Fundus Imaging

Early and accurate diagnosis is crucial for early treatment and good prognosis of ROP. Binocular indirect ophthalmoscopy (BIO) represents the standard technique for screening premature infants.

An alternative method for the ROP screening is digital imaging using wide-angle digital photography of the retina. The most common system for fundus photography is the RetCam imaging system (Clarity Medical Systems, Pleasanton, CA, USA) [25]. High-resolution images can be obtained by a physician or trained non-medical personnel; a pediatric retina/ROP specialist evaluates them. Images are classified according to stage, extent, zone, and presence or absence of “plus” disease (Figure 1) [26,27]. Also the use of a smartphone may offer an exciting method for quick ROP documentation (and further primary referral). Using the phone’s coaxial light source in combination with indirect condensing lens it acts as a simple indirect digital fundus camera [28].

#### 3.5.2. Fluorescein Angiography

Intravenous fluorescein angiography (FAG) is a valuable tool for detailed imaging of the vascular changes, determination of the fovea, and staging in infants with ROP. The RetCam module for FAG can capture the retinal vasculature, the present stage of ROP and plus disease, abnormal vascular patterns in the eyes after spontaneous regression or after therapy, or persistent avascular retina [29,30,31]. Mao et al. reported encouraging results with ultra-wide-angle images of the fundus (including FAG) taken with the Optos 200Tx [32].

#### 3.5.3. Spectral-Domain Optical Coherence Tomography

The development of hand-held spectral-domain optical coherence tomography (SD-OCT) for pediatric use has provided significant insights into human retinal development and allowed the visualization of cross-sectional neurovascular structures of young infants at the cellular level. These features include a shallower foveal pit, persistent inner retinal layers in the fovea, delayed photoreceptor layer development, sub-foveal fluid, and macular edema [33,34,35]. The association of macular edema with either the ROP severity or changes after ROP treatment remains controversial. The presence of macular edema in preterm infants has been associated with poorer visual acuity and neurodevelopmental outcomes [36].

Hand-held SD-OCT imaging is also valuable for clinical evaluation of stage 4 ROP to determine foveal involvement and differentiate between retinal detachment and retinoschisis. Many infants diagnosed with stage 4A ROP suffer retinoschisis without OCT evidence of retinal detachment. This group of infants may represent a sub-stage of the 4A ROP stage, i.e., stage 4A-schisis by SD-OCT [37].

Furthermore, SD-OCT can visualize retinal morphology at the vascular-avascular junction, demonstrate the effect of laser therapy or anti-VEGF therapy, and monitor changes in vivo over time [38].

Fluorescein angiography and SD-OCT may ultimately lead to changes in the definition of ROP and, as a consequence, may serve as a guide for treatment [39].

### 3.6. Artificial Intelligence and Telemedicine

Artificial intelligence represents a current trend for future ROP diagnosis. Improvements in digital imaging have introduced new diagnostic strategies for the diagnosing, monitoring, and treating ROP. Though the standard binocular indirect ophthalmoscopy is considered the gold standard for retinal imaging in infants with ROP, it requires proper and extensive training by qualified and skilled staff. Currently, the wide-angle digital retinal imaging (RetCam) system is extensively used to examine premature neonates with ROP [40].

Applying an automated system based on deep learning (DL) may change the approach to ROP screening and diagnosis in the future. Early detection and timely treatment may halt disease progression at an early stage and prevent the onset of complications [41,42].

Deep learning, a machine learning technology, has been introduced into artificial neural networks. It can automatically classify images and has been applied to ophthalmology for signal processing and imaging of the retina [42,43,44]. DL must be educated with high mathematical precision but can be executed with a lower precision computer. The automatic detection system can be therefore introduced in a general hospital.

Several diagnostic tools based on AI technology have recently been devised to diagnose medical conditions. An ideal deep learning algorithm for ROP must achieve performance comparable with that of ophthalmologists on multidimensional identification of ROP using WFDI. Attallah presented an automated diagnostic tool based on DL techniques to diagnose ROP disease [45]. It extracts significant features by first obtaining spatial features from the four Convolution Neural Networks (CNNs), involving five phases, including ROP images pre-processing, spatial features extraction, spatial-spectral features reduction and extraction, feature integration, and classification phases. The study did not consider the categorization of the ROP disease severity. The diagnostic study developed a cloud-based deep learning platform integrating a multidimension classification and multilevel referral strategy for ROP screening and recommendation. In a published diagnostic multicentric study, a deep learning–based ROP screening platform could identify retinal images using five classifiers: image quality, ROP stages, intraocular hemorrhage, pre-plus/plus disease, and posterior retina with high accuracy [46].

The results of a systematic review and meta-analysis suggest that deep learning models can be instrumental in detecting and classifying ROP, providing high sensitivity, specificity, and repeatability [41]. The results indicate that a cloud-based deep learning platform could identify and classify multidimensional pathological changes of ROP in retinal images with high accuracy and could be suitable for routine ROP screening in general and children’s hospitals. The deep learning platform holds the potential to be applied in neonatal intensive care units, children’s hospitals, and rural primary health care centers for routine ROP screening. In remote areas lacking expertise in ROP, it may be of use combined with telemedicine [41,46].

## 4. Conclusions

Traditional neonatal ophthalmic screening using red reflex test is easy and cheap and it can disclose main congenital ophthalmic pathologies. However, it shows significant weakness in detecting especially retinal disorders. New technologies in imaging (WFDI), hand in hand with telemedicine and artificial intelligence open gates to much more detailed, precise and earlier diagnostics. On the other hand, price, relative system complexity and cumbrous test performance are still limiting factors for wide-spread use.

## Figures and Tables

**Table 1:** Most common diseases detectable with the neonatal ophthalmic screening, approximate incidence and usual timing of treatment.

Anatomical Part of the Eye	Disease Detectable with the Screening	Immaging Technique	Approximate Incidience	Usual Treatment Timing
Anterior segment	dysgenesis of the anterior segment	RRT, WFDI	4:100,000	depends on degree, first months of life
	congenital glaucoma	RRT, WFDI	2–10:100,000	first months of life
	congenital cataract	RRT, WFDI	18–36:100,000	4–8 weeks of life
	uveitis	RRT, WFDI	heterogenous group	according to activity (from immediate to no treatment)
Posterior segment	persistent fetal vasculature	RRT, WFDI	3–7:100,000	depends on degree, 4–8 weeks of life
	vitreous hemorrhage	WFDI; RRT in advanced cases	heterogenous group	depends on degree, within weeks
	uveitis	WFDI; RRT in advanced cases	heterogenous group	according to activity (from immediate to no treatment)
	retinoblastoma	WFDI; RRT in advanced cases	5:100,000	immediate after diagnosis
	retinal/macular hemorrhage	WFDI; RRT in advanced cases	heterogenous group	observation, treatment in indicated cases during weeks
	retinal detachment	WFDI; RRT in advanced cases	rare, heterogenous group	only in selected cases (often impossible to treat)
	Coat´s disease	WFDI; RRT in advanced cases	0.09:100,000	observation, treatment in indicated cases (activity)
	chorioretinal coloboma	WFDI; RRT in advanced cases	5–22:100,000	no treatment required

RRT: red reflex test, WFDI: wide-field digital imaging.
